# A SISO FMCW radar based on inherently frequency scanning antennas for 2-D indoor tracking of multiple subjects

**DOI:** 10.1038/s41598-023-41541-3

**Published:** 2023-10-04

**Authors:** Giulia Sacco, Marco Mercuri, Rainer Hornung, Huib Visser, Ilde Lorato, Stefano Pisa, Guido Dolmans

**Affiliations:** 1 Institut d’Électronique et des Technologies du numéRique (IETR), University of Rennes, UMR CNRS 6164, 35000 Rennes, France; 2 Dipartimento di Informatica, Modellistica, Elettronica e Sistemistica (DIMES), University of Calabria, 87036 Rende, CS Italy; 3 imec-Netherlands, 5656 AE, Eindhoven, The Netherlands; 4 Department of Information Engineering, Electronics and Telecommunications, Sapienza University of Rome, 00184 Rome, Italy

**Keywords:** Electrical and electronic engineering, Design, synthesis and processing

## Abstract

The contextual non-invasive monitoring and tracking of multiple human targets for health and surveillance purposes is an increasingly investigated application. Radars are good candidates, since they are able to remotely monitor people without raising privacy concerns. However, radar systems are typically based on complex architectures involving multiple channels and antennas, such as multiple-input and multiple-output (MIMO) or electronic beam scanning, resulting also in a high power consumption. In contrast with existing technologies, this paper proposes a single-input and single-output (SISO) frequency-modulated continuous wave (FMCW) radar in combination with frequency scanning antennas for tracking multiple subjects in indoor environments. A data processing method is also presented for angular separation and clutter removal. The system was successfully tested in five realistic indoor scenarios involving paired subjects, which were either static or moving along predefined paths varying their range and angular position. In all scenarios, the radar was able to track the targets, reporting a maximum mean absolute error (MAE) of 20 cm and 5.64$^\circ$ in range and angle, respectively. Practical applications arise for ambient assisted living, telemedicine, smart building applications and surveillance.

## Introduction

Radar has been identified as a promising technology for indoor monitoring and healthcare applications^1–18^. Compared to optical and thermal cameras-based devices, radar sensors have the major advantage of not raising privacy concerns while being able to provide fundamental information such as speed, position, shape and health condition^19–23^. Due to the simplicity of their architecture, continuous wave Doppler radars are widely used for vital signs monitoring^24–27^. However, they operate at a fixed frequency and, as a consequence, do not allow to recover information about the target position. To have a range resolution, namely the ability to separate targets in range, a bandwidth is necessary. Therefore, other architectures, such as pulsed ultrawideband (UWB)^28–30^ or frequency-modulated continuous wave (FMCW) radars, should be considered^31–35^. For indoor people monitoring and considering the typical size of human targets, a range resolution of 15–120 cm, corresponding to 1 GHz and 125 MHz bandwidths, is typically considered appropriate^21,36^. In the microwave (below 30 GHz) and millimeter-wave (mmW) ranges, there are several license-free bands that can be used for indoor monitoring^37,38^. Thanks to the lower phase noise and path loss, microwave radars have the ability to monitor subjects at several meters of distance and to penetrate barriers such as glass, doors or walls^39–41^. To this end, the UWB frequency band between approximately 3 and 10 GHz (the exact band depends on the country^42^) is a valid candidate^43,44^.

For people tracking applications, the knowledge of the absolute distance between the radar and the target (i.e., range information) is not enough. On the contrary, it is of the uppermost importance to monitor the 2-D position (range and angle) as a function of time. Contrary to the range resolution, the angular resolution relies on several parameters, namely the antenna beamwidth, the radar architecture and the signal processing chain. Single-input and single-output (SISO) solutions, using only one (omnidirectional or directive) antenna for the transmission and one for the reception, are generally unable to resolve targets in the angular dimension. To solve this issue, a typically adopted solution is to consider a multiple-input and multiple-output (MIMO) UWB architecture, which provides, at the same time, a good range resolution and an angular separation^35,45–47^. The main drawback of this solution is in the complexity of the hardware architecture that requires an array of antennas and multiple channels in transmission and reception, hence demanding a larger silicon area and a higher power consumption. In addition, the single antenna elements have a wide aperture and collect the signal not only in the direction of the target but from the whole environment, including clutter and multipath reflections which deteriorate the quality of the measured data. To reduce the amount of channels and the environmental noise, it is possible to consider a beamforming architecture, that opportunely sets the input amplitudes and phases of the array elements to generate a directive beam in a specified direction^48^. While solving some of the problems of the MIMO, this solution allows to investigate only one direction at a time. When applied in combination with FMCW radar, for each chirp with a duration *T*, the system can detect only one angular direction. Therefore, to have a complete map of the environment, it is necessary to consider a time interval $N_{\text {a}}\times T$, with $N_{\text {a}}$ being the number of investigated angles.

**Figure 1:**
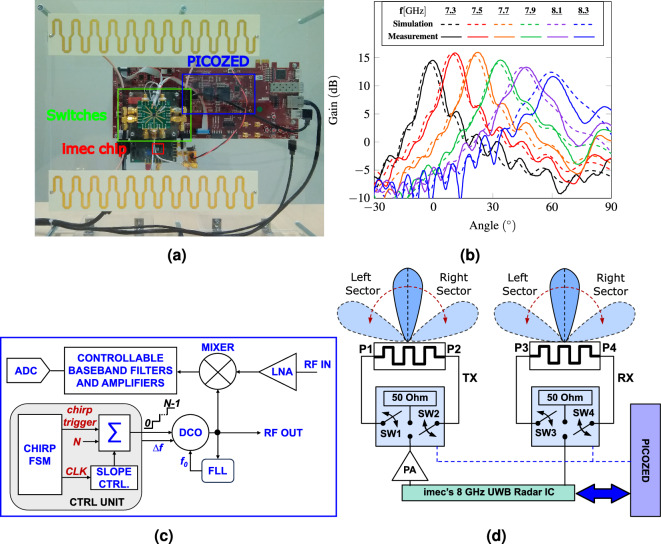
(**a**) Photo of the radar with the frequency scanning antennas, (**b**) radiation patterns as a function of frequency, (**c**) block diagram of the imec’s 8 GHz UWB radar IC, and (**d**) block diagram of the radar system.

To simplify the system architecture and reduce the consumption, while being able to obtain 2-D information, in this work, we propose a SISO FMCW radar sensor integrating two inherently frequency scanning antennas for the concurrent indoor tracking of multiple human subjects. This solution only requires two channels and two antennas, one for transmission and one for reception. As a consequence, the chip silicon area as well as the power consumption is smaller, compared to what is required for an electronically scanning MIMO system and does not need any complex beamformers. Contrary to MIMO, the investigation of the different angular directions is operated by the reorientation of the main antenna beam due to the variation in frequency. As an additional benefit of this architecture, the receiving and transmitting antennas, thanks to their directivity illuminate one angular sector at the time. While this does not eliminate the problem of ghosts, it highly reduces their presence since the collected signal comes only from the angle illuminated by the beam and it is not affected by the presence of other targets in the remaining portion of the environment that may degrade the collected signal due to multipath. The concept of using a radar with frequency scanning antennas was already discussed in^49^, where the attention was focused on the simultaneous vital signs monitoring of multiple static targets. To our best knowledge, nothing was reported for people tracking, which is the core application of this work. This requires to propose a detection and tracking algorithm for angular separation and clutter removal specifically developed for the proposed system and to validate it experimentally on human subjects in multiple scenarios. The system architecture, together with the proposed algorithm are expected to introduce a significant improvement in indoor monitoring systems compared to conventional solutions, by reducing the size, the power consumption, and the system complexity.

## Materials and methods

### Materials

#### System hardware

A photo and a block diagram of the radar system are reported in Fig. 1a–d, respectively.

It is based on the imec’s 8 GHz UWB radar integrated circuit (IC), which integrates a digital linear discrete frequency-modulated continuous wave (LD-FMCW) architecture, which is essentially a linear FMCW with the benefit of the digital implementation^50–52^. The radar is designed to operate in the 7.3–8.3 GHz range and is compliant with the worldwide indoor UWB spectrum regulations^42^. The block diagram is show in Fig. 1c. The chip is fabricated in 40 nm complementary metal-oxide semiconductor (CMOS) technology and integrates a digital controlled oscillator (DCO), a low-noise amplifier (LNA), a mixer, a baseband filter and amplifier, and a 9-bit analog-to-digital converter (ADC) with sampling frequency of 12.5 MHz. The main unit of the chip is the control unit implemented by a finite-state-machine (FSM), a slope controller and an accumulator ($\Sigma$). This allows generating the LD-FMCW chirp, which consists of a group of N continous wave (CW) pulses, each of Ts seconds, whose frequencies are increased from pulse to pulse by a fixed frequency increment $\Delta f$, always ensuring phase continuity among pulses. Therefore, the frequency vs. time response of LD-FMCW chirp presents a staircase shape trend. A frequency-locked loop (FLL) sets the DCO to operate at the initial frequency $f_0$. A new chirp is generated every time a new trigger is provided to the accumulator by the FSM. The slope controller counts the number of cycles of the master clock (CLK) and sends a trigger to the accumulator when a number of clock periods equal to Ts is passed. The accumulator provides a code to the DCO corresponding to the n-th frequency increment. Once the chirp has been generated, the accumulator is reset and the DCO start again from $f_0$. The default waveform parameters are: $f_0$ = 7.3 ﻿GHz, Ts = 80 ns, N = 512, $\Delta f = {1.95} \,{\hbox {MHz}}$, although they are tunable over wide ranges. The chip is mounted on a PCB which is connected to the PicoZed7030 through a PicoZed Carrier Card V2. The latter reads the data from the chip, performs pre-processing (i.e., digital filtering) and finally sends the data to a laptop. The transmitted signal is amplified by the Mini-Circuits ZX60-06203LN+ amplifier^53^. The chirp duration *T* is 40.96 ${\upmu {\hbox {s}}}$, sufficiently short to assume the target being motionless within this interval, while the pulse repetition interval (PRI) is 1.3 ms, which satisfies the Nyquist theorem for typical human speeds. This corresponds to a duty cycle of about 3$\%$. During the remaining period (about 97%), no signal is transmitted and most of the circuit blocks in the chip are switched-off. Considering this and setting the transmitted power lower to − 6 dBm, the chip consumes only 680 ${{\upmu }\hbox {W}}$ (average power)^50^.

The radar is connected to two rampart line antennas (Fig. 1a). Details on the antenna design can be found in^54^. Each antenna has two ports and can re-orientate the main beam direction according to the frequency of the feeding signal (Fig. 1d). When P1 and P3 are connected to the electromagnetic (EM) source, through the switches’ ports SW1 and SW3, and P2 and P4 are connected to a matched load (50 $\Omega$), through SW2 and SW4, the antenna beam direction varies from 0$^\circ$ to 60$^\circ$, scanning the right sector. By using the switches to invert the feed with the load, the antenna scan from − 60$^\circ$ to 0$^\circ$, corresponding to the left sector. With this last expedient, with only 2 chirps, a spatial coverage of 120$^\circ$ is ensured. The switches are controlled by the PicoZed7030. The simulated and measured radiation patterns are reported in Fig. 1b for 6 directions in the right sector.

#### Link budget

The operation principle to track a subject in the room is to detect their Doppler information. Hence, the main challenge is to detect the phase shift due to the target motion. The root mean square (RMS) phase noise on a carrier frequency is directly proportional to the thermal noise at the input and the noise figure of the receiver^55^. It can be expressed as:

1 $$\begin{aligned} \Delta \Phi _{\text {RMS}}=\sqrt{\frac{FkT_{\text {e}}B_{\text {w}}}{P_{\text {re}}}} \end{aligned}$$

where *F* is the noise figure of the receiver, *k* is the Boltzmann’s constant, $T_{\text {e}}$ is the temperature, $B_{\text {w}}$ is the instantaneous baseband receiver bandwidth, while $P_{\text {re}}$ is the power of the received signal, which can be expressed as^55^:

2 $$\begin{aligned} P_{\text {re}}=\frac{P_{\text {tr}} G_{\text {t}} G_{\text {r}} \lambda _0^2 \sigma \Gamma }{(4\pi )^2 D^4 L_{\text {s}}} \end{aligned}$$

where $P_{\text {tr}}$ is the power of the transmitted signal, $G_{\text {t}}$ and $G_{\text {r}}$ are respectively the transmitting and receiving antennas’ gains, $\sigma$ is the radar cross section (RCS), $\Gamma$ is the reflection coefficient at the air/skin interface, *D* is the target’s absolute distance, and $L_{\text {s}}$ takes into the account all the system losses. In this analysis, it was considered $\sigma = {0}\,\hbox {dBsm}$ as the RCS of the person, $\Gamma = 0.7$, $kT_{\text {e}} = {-174}\,\hbox {dBm}$, and $D = {5}\,{\hbox {m}}$ as maximum range. In addition, the chip has the following parameters: $P_{\text {tr}} = {-6}\,\hbox {dBm}$, $G_{\text {t}}= G_{\text {r}} = {15}\,\hbox {dBi}$, $\lambda _0 = {0.0373}\,\hbox {m}$, $F = {12.5}\,{\hbox {dB}}$, $L_{\text {s}} = {2}\,\hbox {dB}$, $f_{\text {ADC}} = {12.5}\,\hbox {MHz}$, $K = 512$ samples per chirp ($T = {40.96}\,{\upmu }\hbox {s}$), and $B_{\text {w}} = {1.7}\,{\hbox {MHz}}$. In this application, the signal of interest occupies a bandwidth smaller than the maximum Nyquist bandwidth. In fact, performing a K-point FFT over the acquired waveform to extract information about a particular frequency component, is equivalent to digitally filter the signal with a bandwidth equal to the frequency resolution of the FFT, namely $B_{\text {w}} = f_{\text {ADC}}/K \approx {24.4}\,\hbox {kHz}$. Considering a subject walking of 10 cm around the nominal distance of 5 m, the corresponding RMS Doppler shift is about 30.58 rad, while the estimated RMS phase noises is 0.035 rad (Eq. 1). This results in an signal-to-noise-ratio (SNR) of about 45 dB. Therefore, by this link budget analysis, the chip is able to properly track a subject within typical room settings.

**Figure 2:**
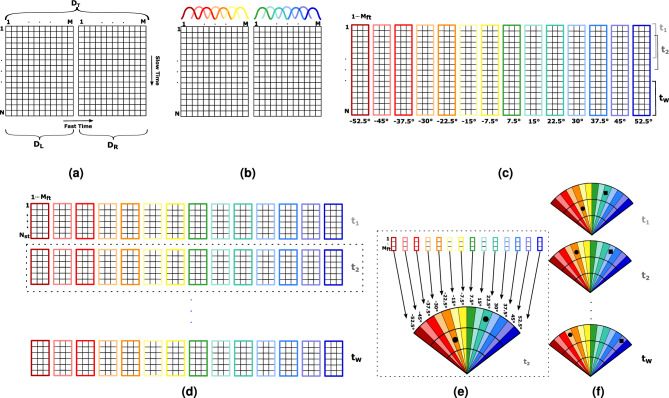
Schematic representation of the signal processing chain: (**a**) data acquisition and organization in the $D_{\text {T}}$ matrix, (**b**) windowing in *fast time*, (**c**) windowing in *slow time*, (**d**) sub-matrices resulting from the windowing process to which FFT and STD are applied, (**e**) organization of the STD vectors in a 2-D map, and (**f**) target tracking.

### Methods

#### Data collection

The digitized demodulated radar signals are arranged in the $D_{\text {T}}$ matrix consisting of two sub-matrices, one corresponding to the left sector $D_{\text {L}}$ and the other to the right sector $D_{\text {R}}$ (see Fig. 2a). Each row of these sub-matrices contains $M=512$ elements, sampled in *fast time* every 80 ns. The sampling interval in *slow time* is the double of the PRI (of ${1.3}\,{\hbox {ms}}$) and corresponds to 2.6 ms (i.e., 1.3 ms for the acquisition of the *m*-th row of $D_{\text {L}}$ and 1.3 ms for the acquisition of the *m*-th row of $D_{\text {R}}$), while the number of rows *N* depends on the measurement duration.

#### Position estimation and clutter removal

Since the main beam direction varies according to the frequency, each target is illuminated by the EM radiation for a duration $t_a-t_b<T$. Given that the antenna of the proposed system is highly directive, when the beam is pointing at a specific direction only the reflection resulting from that direction will be collected by the radar, filtering the multipath that may result from the presence of clutter and/or targets positioned in other portions of the environment. This also implies that the information about a target is encoded only in a limited number of adjacent columns, corresponding to a specific angular sector, whose samples were collected during the time interval $t_a<t<t_b$. From a mathematical point of view and for one target, this can be expressed as

3 $$\begin{aligned} D_{\text {Target}} = {\left\{ \begin{array}{ll} A\sin \left[ 2\pi \left( \frac{B}{T}\frac{2d}{c}t+f_0\frac{2d}{c}\right) \right] &{} \hbox { if}\ t_a<t<t_b\\ 0 &{} \text {otherwise} \end{array}\right. } , \end{aligned}$$

where *c* is the speed of light in free space, *A* is the voltage amplitude, *d* is the target’s absolute distance, while *B* is the bandwidth of the chirp, respectively.

To obtain an angular separation, $D_{\text {T}}$ is divided in 30 sub-matrices $N \times M_{\text {ft}}$ (with $M_{\text {ft}}=64$), using a windowing operation in *fast time* (Fig. 2b). An overlap of 32 samples is considered to ensure an angular scanning step of 3.75$^\circ$. Therefore, if a subject is found in a given angular sector, its angular position is set to the center value of the corresponding sector, namely at ${\pm \, 3.75}^\circ$, ${\pm \, 7.5}^\circ$, ${\pm \, 11.25}^\circ$, ${\pm \, 15}^\circ$, ${\pm \, 18.75}^\circ$, ${\pm \, 22.5}^\circ$, ${\pm \, 26.25}^\circ$, ${\pm \, 30}^\circ$, ${\pm \, 33.75}^\circ$, ${\pm \, 37.5}^\circ$, ${\pm \, 41.25}^\circ$, ${\pm \, 45}^\circ$, ${\pm \, 48.75}^\circ$, ${\pm \, 52.5}^\circ$, and ${\pm \, 56.25}^\circ$. For each sub-matrix, each row is then multiplied with a Hanning window (Fig. 2b). It is worth noticing that, the range resolution *R* in an FMCW radar, intended as the possibility to separate two adjacent targets at different ranges, is associated to the bandwidth according to

4 $$\begin{aligned} R=\frac{c}{2B}\,. \end{aligned}$$

While with a 1 GHz bandwidth $R=$ 15 cm, this value is increased to 1.2 m with the windowing in *fast time* (a window of 64 samples corresponds to a band of 125 MHz). The angular resolution is also depending on the dimension of the windows in *fast time*, and two targets at the same range will be resolved angularly only if they are located in different angular sectors. Considering the typical size of a human target and of a room, and making a compromise between angular and range resolution, the dimension of the angular sectors is fixed to 7.5$^\circ$.

Since the targets are moving, to monitor the variation of their position, in addition to the Hanning windows in *fast time*, rectangular windows in *slow time* are used to divide the acquisition in multiple time frames ($t_1,t_2,\ldots ,t_{\text {W}}$ in Fig. 2c). During each frame, composed of $N_{\text {st}}=400$ rows (corresponding to about 1 s), the targets are assumed to remain within the same range bin of 1.2 m. The overlap of the windows in *slow time* is of 200 rows. The dimensions of the sub-matrices resulting from the windowing process in *fast* and *slow time* are $N_{\text {st}}\times M_{\text {ft}}$ (Fig. 2d).

For each one of these $N_{\text {st}}\times M_{\text {ft}}$ sub-matrices, we first perform the fast Fourier transform (FFT) in *fast time* and then we determine the standard deviation (STD) in *slow time*. These operations result in a vector of $1\times M_{\text {ft}}$. The STD is used to distinguish the moving subjects from stationary reflectors (e.g., furniture, objects, clutter, etc.). While the contribution of the static objects on the signal collected by the radar remains the same for all the chirps, the human targets’ motions will result in a Doppler signal that will be varying in *slow time*. This implies that, while an almost null STD will correspond to all the clutter elements, the STD will be maximized in all the range/angular positions corresponding to the location of human targets. This is also valid for static subjects, who can still be identified by exploiting the Doppler signal induced by the cardiopulmonary activity^49^. Since each vector is relative to one angular sector, the STD is applied for each time frame to the 30 sub-matrices. This allows to create the 2-D map of the room at a give time frame (Fig. 2e).

#### Tracking

For each time frame, local maxima corresponding to the detected targets are isolated and a tracking algorithm is used to relate the points in different time frames and define the trajectory of targets (Fig. 2f). A noise threshold was set and only the local maxima with an amplitude of at least the half of the peak amplitude detected in the frame were considered. If multiple targets in a 0.3 m or 3.75$^\circ$ distance were detected, an equivalent target was placed in the middle of the points. Two maxima belonging to two consecutive frames are considered as the same target if their ranges *r* and angular positions *a* at the frames *n* and $n+1$ respect the following condition;

5a $$\begin{aligned} \left| r_{\text {n}}-r_{\text {n+1}}\right|&\le r_{\text {t}}\,,\end{aligned}$$

5b $$\begin{aligned} \left| a_{\text {n}}-a_{\text {n+1}}\right|&\le a_{\text {t}}\,, \end{aligned}$$

where $r_{\text {t}}$ and $a_{\text {t}}$ are the accepted tolerances in terms of range and angle, that correspond to 1.5 m and 15$^\circ$. These values were chosen in accordance with the average sizes of human beings and of indoor environments.

## Results and discussion

### Experimental validation

All procedures in this study protocol adhered to the ethical principles of the Declaration of Helsinki. Written informed consent was provided by all patients for data collection and picture publication before they were enrolled in the study. The Imec Netherlands Medical Ethical Committee (INMEC) reviewed and approved the study protocols (IP-19-WATS-TIP2-056). All the collected data were pseudonymized. The experiments have been conducted in a $6\times {7}\,{\hbox {m}^2}$ room environment which has a steel-reinforced concrete floor, metal wall parts and a metal tiles. The radar was positioned on a table at about 1 m of height.

**Figure 3:**
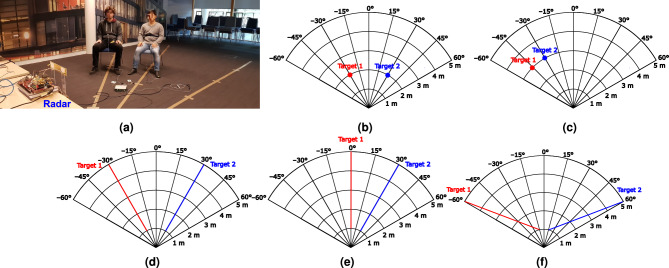
(**a**) Measurement set-up and considered scenarios (**b**) two static targets at ${-30}^\circ$ and ${30}^\circ$ at 2 m, (**c**) two static targets at ${-45}^\circ$ and ${-30}^\circ$ at 3 m, (**d**) two targets at ${-30}^\circ$ and ${30}^\circ$ moving from 1 to ${5}\,{\hbox {m}}$, (**e**) two targets at ${0}^\circ$ and ${30}^\circ$ moving from 1 to ${5}\,{\hbox {m}}$, and (**f**) two targets moving from ${1}\,{\hbox {m}}$ and ${\pm \, 15}^\circ$ to ${5}\,{\hbox {m}}$ and ${\pm \, 60}^\circ$.

The described algorithm is tested on the 5 scenarios and the set-up measurement illustrated in Fig. 3: two static targets at ${-30}^\circ$ and ${30}^\circ$ at 2 m; two static targets at ${-45}^\circ$ and ${-30}^\circ$ at 3 m; two targets at ${-30}^\circ$ and ${30}^\circ$ moving back and forth from 1 to ${5}\,{\hbox {m}}$; two targets at ${0}^\circ$ and ${30}^\circ$ moving from 1 to ${5}\,{\hbox {m}}$; two targets moving from ${1}\,{\hbox {m}}$ and ${\pm \, 15}^\circ$ to ${5}\,{\hbox {m}}$ and ${\pm \, 60}^\circ$. In this validation, we considered four subjects differing in height (155–180 cm) and build. In all the scenarios, only two targets at a time were measured as an example, but there is no theoretical limit in the number of people that can be simultaneously tracked. However, if the number of targets increases, the effect of multipath is expected to increase but to remain lower than the one that would exist in a MIMO system thanks to the antenna directivity.

To guide the volunteers during the experiments, some marks on the floor were used to reproduce the patterns reported in Fig. 3 . Depending on the scenario, the targets had either to remain in a fixed point or to move along specified path. The target position (range and angle) was collected for each chirp. The error in range was computed for every measurement point for the static case (scenarios 1 and 2), and only for the extreme values of the range for the remaining scenarios. For the angle, the error estimation was obtained for all the points for the static configurations (scenarios 1 and 2) and the measurements where the targets were moving at a fixed angle (scenarios 3 and 4). For the measurements where both range and angle were varying (scenario 5), the error in angle as well as the one in range were evaluated only at the extreme points of the path.

For the first two scenarios, two static configurations were analysed to prove that the target detection algorithm, based on the use of the STD is able to retrieve the position of static human targets. The proposed solutions could accurately reconstruct the positions for two targets at the same range but in different angle positioned symmetrically with respect to the origin (scenario 1) or in almost adjacent angular sectors (scenario 2). The absolute errors in distance and angle estimation reach 0.35 m and 7.5$^\circ$ in scenario 1, and 0.6 m and 7.5$^\circ$ in scenario 2. These values are higher for scenario 2 probably because of the multipath that is not completely rejected for two targets that are in close proximity, but they are still lower than the system resolution.

**Figure 4:**
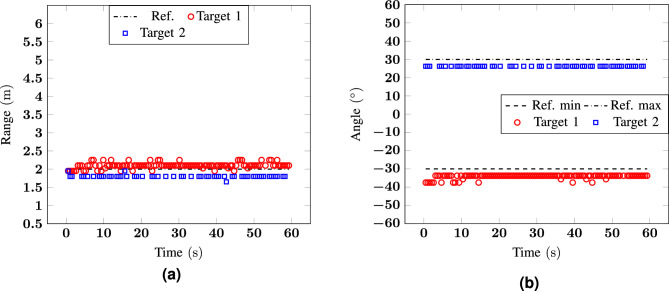
Measurement 1: (**a**) range and (**b**) angle as a function of time for a scenario with two targets at ${-30}^\circ$ and ${30}^\circ$ at ${2}\,{\hbox {m}}$.

**Figure 5:**
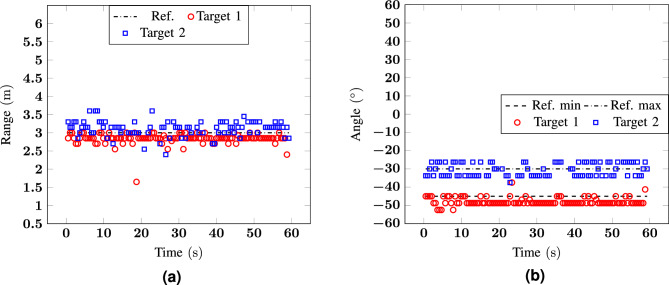
Measurement 2: (**a**) range and (**b**) angle as a function of time for a scenario with two targets at ${-45}^\circ$ and ${-30}^\circ$ at 3 m.

**Figure 6:**
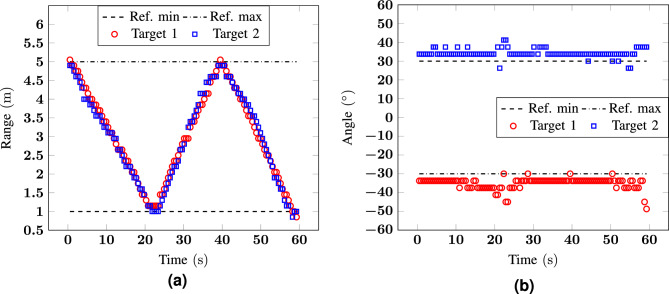
Measurement 3: (**a**) range and (**b**) angle as a function of time for a scenario with two targets at ${-30}^\circ$ and ${30}^\circ$ moving from 1 to ${5}\,{\hbox {m}}$ (low speed).

**Figure 7:**
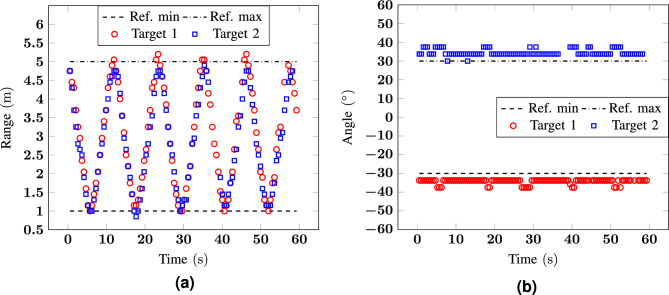
Measurement 4: (**a**) range and (**b** ) angle as a function of time for a scenario with two targets at ${-30}^\circ$ and ${30}^\circ$ moving from 1 to ${5}\,{\hbox {m}}$ (high speed).

For the third scenario, 2 different configurations are analysed: in the first measurement, the two volunteers are moving at a slow speed of about 0.2 ${\hbox {m/s}}$, while in the second measurement their speed is four times higher. This analysis was done to test the algorithm in more controlled (0.2 ${\hbox {m/s}}$) and in more realistic (0.8 ${\hbox {m/s}}$) conditions, to verify how the errors in range and angle are affected by the speed. From the results shown in Figs. 6 and 7, it is already possible to conclude that, in both configurations, the errors are comparable and lower than 0.25 m for the range and 10$^\circ$ for the angle. Considering the typical dimensions of a human being, these errors are acceptable and the measurements give an accurate positioning of the targets. A more detailed statistical analysis of the error for these and the following measurement configurations is reported in “Statistical analysis”.

**Figure 8:**
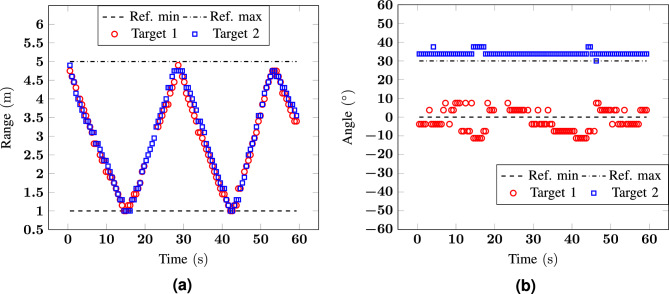
Measurement 5: (**a**) range and (**b**) angle as a function of time for a scenario with two targets at ${0}^\circ$ and ${30}^\circ$ moving from 1 to ${5}\,{\hbox {m}}$.

**Figure 9:**
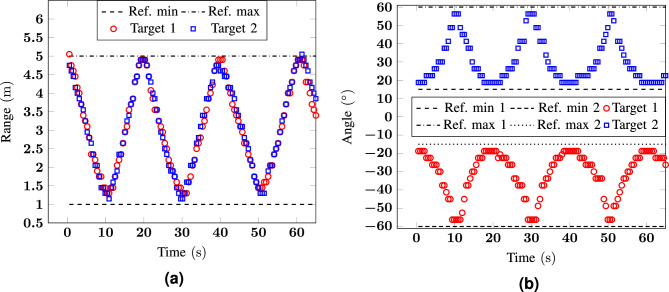
Measurement 6: (**a**) range and (**b**) angle as a function of time for a scenario with two targets moving from 1 and ${\pm \, 15}^\circ$ to ${5}\,{\hbox {m}}$ and ${\pm \, 60}^\circ$.

As fourth scenario, one of the two targets is moving at ${30}^\circ$ from 1 to ${5}\,{\hbox {m}}$, while the second is walking in the same range at ${0}^\circ$. The difference between this scenario and the one shown before is given by the fact that, for the way the radar system is implemented, there is no angular sector centered at 0$^\circ$. As a consequence, a target at 0$^\circ$ will be placed either at − 3.75$^\circ$ or at 3.75$^\circ$. The results of the range and angular information over time are shown in Fig. 8. While also in this case the error in range stays lower than 0.25 m, the error in angle increases up to about 15$^\circ$ for some measurement points. However, it is worth noticing that the error in angle is the highest when the targets are at in proximity of the radar (approximatively at 1 m). At this distance, the thorax width is comparable with the angular sector dimension, thus the subject may cross multiple angular sectors. In such a situation, a higher error can be tolerated.

As a final scenario, the two volunteers were concurrently walking in range, from 1 to ${5}\,{\hbox {m}}$, and in the angular dimension, from ${\pm \, 15}^\circ$ to ${\pm \, 60}^\circ$. Also in this case, the reported errors are comparable with the ones of Figs. 6 and 7.

### Statistical analysis

To analyze the data statistically, the mean absolute error (MAE) and the root-mean-square error (RMSE) have been considered:

6a $$\begin{aligned} MAE&=\frac{1}{N_{\text {elem}}}\sum _{i=1}^{N_{\text {elem}}}\left| x-x_{\text {ref}}\right| \end{aligned}$$

6b $$\begin{aligned} RMSE&=\sqrt{\frac{1}{N_{\text {elem}}}\sum _{i=1}^{N_{\text {elem}}}\left( x-x_{\text {ref}}\right) ^2}\,, \end{aligned}$$

where $N_{\text {elem}}$ is the number of measurement points for which the target position *x*, corresponding either to the angle or the range, is known and can be compared to a reference value $x_{\text {ref}}$. The obtained results for the four measured scenarios illustrated in Figs. 4, 5, 6, 7, 8 and 9 are listed in Table 1.

**Table 1:** MAE and RMSE in the four measured scenarios.

Measurement	MAE				RMSE			
	Range (m)		Angle ($^\circ$)		Range (m)		Angle ($^\circ$)	
	**Target 1**	**Target 2**	**Target 1**	**Target 2**	**Target 1**	**Target 2**	**Target 1**	**Target 2**
1	0.11	0.20	4.22	3.75	0.13	0.20	4.38	3.75
2	0.16	0.19	3.07	3.23	0.22	0.24	3.56	3.52
3	0.17	0.09	4.48	4.27	0.19	0.12	4.78	4.46
4	0.07	0.10	4.39	5.03	0.09	0.12	4.75	5.80
5	0.12	0.13	5.64	4.01	0.17	0.18	6.19	4.16
6	0.20	0.19	3.78	3.75	0.23	0.21	3.24	2.50

The MAE stays between 7 and 20 cm for the range and between 3.07$^\circ$ and 5.64$^\circ$ for the angle. The worst case scenario is registered for the range of target 2 in measurement 1, and target 1 in measurement 6, while for the angle of target 1, in Measurement 5, respectively. The highest error in angle is for the target moving at 0$^\circ$. The reason was explained in  “Experimental validation”. However, also for this measurement condition, the error is only a few degrees higher than the one reported in the other scenarios. When considering the same scenario but different speeds (e.g., in measurement 3 and 4) there are only negligible differences. The MAE varies between 0.07 and 0.17 m with better results for the cases where the targets were moving at a higher speed. The MAE in angle is at most 5.03$^\circ$ and the difference for the two considered speeds is of about 1$^\circ$ with slight better results for measurement 3. Similar findings are found also from the RMSE that corresponds to 0.23 cm, for target 1 in Measurement 6, and to 6.19$^\circ$, for target 1 in Measurement 5. It is worth noticing that, even with the range resolution reduction due to the windowing from 15 to 120 cm, the resulting errors are perfectly fine for indoor localization and tracking purposes. Since the presented system is scalable in frequency, for all the applications where a higher range resolution is required it is possible to consider either a wider portion of the unlicensed frequency band between 3 and 10 GHz or to move to mmW and consider the unlicensed band around 60 GHz.

The obtained results have then been compared with other radar systems proposed in the literature (Table 2).

**Table 2:** Comparison of tracking errors among different radar technologies.

	Radar technology	Frequency	**Error formula**	Error	
				**Range (m)**	Angle ($^\circ$)
This work	SISO FMCW	7.3–8.3 GHz	RMSE	0.09–0.24 m	2.50–6.19$^\circ$
^14^	Dual-frequency continuous-wave	2.4 GHz, 2.41 GHz	RMSE	0.11–0.58 m	−
^15^	Impulse-radio UWB	3.1–5.6 GHz	Averaged RMSE	0.10–0.13 m	−
^16^	Wi-Fi-based multi-antenna passive	80 MHz around 2.45 GHz	Averaged RMSE	0.32 m	−
^17^	MIMO FMCW s	24.025–24.225 GHz	Absolute error	$\le$0.25 m	$\le$ 10$^\circ$

As it is possible to see, the errors reported in this work are comparable or even smaller than the ones obtained with alternative radar solutions. Moreover, the proposed system has the major advantage of a simpler hardware (SISO) and of a lower power consumption.

## Conclusions

To answer to the increasing need for non-invasive systems for indoor localization and tracking of multiple targets for health and surveillance purposes, this paper proposes a SISO FMCW radar used in combination with frequency scanning antennas. The radar architecture requires only one channel for the transmission and another one for the reception, ensuring a significant reduction of the power consumption compared to other commonly proposed solutions (e.g., MIMO or electrical beamsteering). The system, integrating only two antennas, was proved to resolve in the range and angle dimensions, paired targets while moving within the same environment. To achieve the angular separation of the targets, a tracking algorithm was developed. The use of the STD in *slow time* in combination with the high antenna gain (between 10 and 15 dBi) were proposed as powerful tools to reduce the effect of the environmental clutter to a negligible level.

The system together with the algorithm were tested in five different scenarios: two static targets at ${-30}^\circ$ and ${30}^\circ$ at 2 m; two static targets at ${-45}^\circ$ and ${-30}^\circ$ at 3 m; two targets at ${-30}^\circ$ and ${30}^\circ$ moving from 1 to ${5}\,{\hbox {m}}$; two targets at ${0}^\circ$ and ${30}^\circ$ moving from 1 to ${5}\,{\hbox {m}}$; and two targets moving from ${1}\,{\hbox {m}}$ and ${\pm \, 15}^\circ$ to ${5}\,{\hbox {m}}$ and ${\pm \, 60}^\circ$. The highest registered MAE is 20 cm for the range and 5.64$^\circ$ for the angular measurements, while the RMSE error is at most 23 cm for the range and 6.19$^\circ$ for the angle. These values are perfectly acceptable for the proposed application. The obtained errors are also comparable or outperforming current state-of-the-art alternatives.

## Data Availability

The data that support the plots within this paper and other findings of this study are available from the corresponding author upon reasonable request.
